# Differentially Expressed miRNAs in Ewing Sarcoma Compared to Mesenchymal Stem Cells: Low miR-31 Expression with Effects on Proliferation and Invasion

**DOI:** 10.1371/journal.pone.0093067

**Published:** 2014-03-25

**Authors:** Bianca Karnuth, Nicolas Dedy, Tilmann Spieker, Elizabeth R. Lawlor, Stefan Gattenlöhner, Andreas Ranft, Uta Dirksen, Heribert Jürgens, Andreas Bräuninger

**Affiliations:** 1 Gerhard-Domagk-Institute for Pathology, University Hospital Münster, Westfälische Wilhelms-University, Münster, Germany; 2 Department of Orthopedics and Tumor Orthopedics, University Hospital Münster, Westfälische Wilhelms-University, Münster, Germany; 3 Departments of Pediatrics and Pathology, University of Michigan, Ann Arbor, Michigan, United States of America; 4 Department of Pathology, Justus-Liebig-University, Giessen, Germany; 5 Pediatric Hematology and Oncology, University Hospital Münster, Westfälische Wilhelms-University, Münster, Germany; University Hospital of Modena and Reggio Emilia, Italy

## Abstract

Ewing sarcoma, the second most common bone tumor in children and young adults, is an aggressive malignancy with a strong potential to metastasize. Ewing sarcoma is characterised by translocations encoding fusion transcription factors with an EWSR1 transactivation domain fused to an ETS family DNA binding domain. microRNAs are post-transcriptional regulators of gene expression and aberrantly expressed microRNAs have been identified as tumor suppressors or oncogenes in most cancer types. To identify potential oncogenic and tumor suppressor microRNAs in Ewing sarcoma, we determined and compared the expression of 377 microRNAs in 40 Ewing sarcoma biopsies, 6 Ewing sarcoma cell lines and mesenchymal stem cells, the putative cellular origin of Ewing sarcoma, from 6 healthy donors. Of the 35 differentially expressed microRNAs identified (fold change >4 and q<0.05), 19 were higher and 16 lower expressed in Ewing sarcoma. In comparisons between Ewing sarcoma samples with EWS-FLI or EWS-ERG translocations, with differing dissemination characteristics and of primary samples and metastases no significantly differential expressed microRNAs were detected using various stringency criteria. For miR-31, the microRNA with lowest expression in comparison to mesenchymal stem cells, functional analyses were performed to determine its potential as a tumor suppressor in Ewing sarcoma. Two of four miR-31 transfected Ewing sarcoma cell lines showed a significantly reduced proliferation (19% and 33% reduction) due to increased apoptosis in one and increased length of G1-phase in the other cell line. All three tested miR-31 transfected Ewing sarcoma cell lines showed significantly reduced invasiveness (56% to 71% reduction). In summary, we identified 35 microRNAs differentially expressed in Ewing sarcoma and demonstrate that miR-31 affects proliferation and invasion of Ewing sarcoma cell lines in ex vivo assays.

## Introduction

Ewing sarcoma (ES) is the second most frequent bone tumor in children and young adults with an overall incidence of about 1.3 cases per million people [1], [2]. The histochemically characterised small, blue round cell tumor is highly aggressive with a distinct propensity for dissemination [3], [4]. Despite significant progress in treating Ewing sarcoma over the last years, the prognosis of the 20% of patients with primary disseminated disease remains poor, with an event free survival of less than 20% [5].

The typical genomic aberration in ES is a translocation between the *EWSR1* gene and an ETS-family member with *FLI1* in 85% and *ERG* in 5–10% of cases. In the resulting fusion protein the transactivation domain of EWS is combined with the DNA-binding domains of FLI1 or ERG to create an aberrant transcription factor [6], [7], which effects the expression of more than 1000 genes [8], [9].

Currently most evidence indicates that mesenchymal stem cells (MSCs) are the progenitors of ES. In ES cell lines EWS-FLI1 knockdown results in a MSC-like gene expression pattern and expression of EWS-FLI1 in heterologous cell types has shown that only MSCs of either mesodermal or neural crest origin are permissive for EWS-FLI1 [10]–[13]. Importantly, although EWS-FLI can induce malignant transformation of murine MSCs, it is by itself insufficient to transform human stem cells indicating that other cooperating events are required [11], [13].

microRNAs (miRNAs) are 18–25 nucleotide long non-coding RNA that act as post-transcriptional regulators of gene expression by hybridizing to complementary target-mRNA regions causing inhibition of translation with or without degradation of the mRNA. Today it is assumed that there are more than 1500 miRNAs which affect the expression of over 60% of human genes [14], [15]. Over the last years aberrantly expressed miRNAs were identified in most tumor types and for several of those an important role in tumor pathogenesis and metastasis could be demonstrated [16], [17].

Recently the roles of miRNAs in ES were analysed in several studies. These were either focussed on the detection of miRNAs regulated by EWS-FLI1 in ES cell lines [18]–[22], or on the identification of prognostic miRNAs by comparison of ES with different clinical course or the detection of miRNAs specifically related to ES stem cells [23]–[25].

To identify differentially expressed miRNA relevant for ES pathogenesis and clinical behaviour, including also miRNAs affected by events other than EWS-FLI, we used a different experimental approach. We generated miRNA expression profiles of 377 highly characterized miRNAs of the more than 1500 miRNAs for 40 fresh-frozen ES samples, including primary cases and metastases, cases with different translocation types and six ES cell lines and compared these to those of MSCs from six healthy donors as the putative cells of origin. For miR-31, which is the miRNA with lowest expression in all ES samples compared to MSC samples, we demonstrate effects on proliferation and invasion of ES cell lines.

## Materials and Methods

### Ethics Statement

All ES samples were taken from patients registered in the EICESS-92 study or in the EURO-E.W.I.N.G 99 study who gave informed written consent for usage of biopsy material not necessary for diagnostic purposes for scientific purposes, in accordance with the Declaration of Helsinki and approval of the Ethics commission (Ethics commission of the Medical Faculty of the Westfalian-Wilhelms University Münster). Human bone marrow MSCs were isolated from femoral heads or femoral condyle cancellous bone of patients undergoing hip or knee replacement surgery with informed written consent of the patients and approval of the local Ethics commission (Ethics commission of the Medical Faculty of the Westfalian-Wilhelms University Münster).

### Patient Samples

ES fresh frozen samples (40 samples for TaqMan Low Density Array (TLDA) analysis and further 10 samples for validation) used for the miRNA analysis were taken from patients registered in the EICESS-92 study or in the EURO-E.W.I.N.G 99 study and preserved in the Gerhard-Domagk-Institute for Pathology [26], [27]. Only samples with at least 80% tumor cell content were used.

### MSCs, Cell Lines and Cell Culture

Human bone marrow MSCs were isolated from femoral heads or femoral condyle cancellous bone of patients undergoing hip or knee replacement surgery (six samples for the TLDA and four further samples for verification). Recovered bone marrow was diluted in PBS with 2 mM EDTA. After cell suspensions were filtered though a 100 μm membrane, mononuclear cells were enriched using a Ficoll-Paque PLUS gradient (GE Healthcare Life Sciences, Uppsala, Sweden). MSCs were then separated by magnetic cell sorting using CD271-micro beads (Miltenyi Biotec, Bergisch Gladbach, Germany) and cultured in NH Expansion Medium for about 20 days (Miltenyi Biotec). A detailed characterisation of the MSCs is given in Dataset S1. It should, however, be noted that the *in vitro* expansion and the derivation of the MSCs from adults could be a source of misinterpretations in comparisons with ES.

ES (TC-71, CADO-ES, WE-68, RD-ES) and osteosarcoma (OS)(HOS, MG-63) cell lines, HEK293T and MCF-7 (breast cancer) cell lines were cultured in RPMI-1640 containing 10% fetal calf serum (FCS) (Life Technologies, Carlsbad, CA, USA) and 1% penicillin/streptomycin (PAA, Pasching, Austria). Cell line MDA-MB-231 (breast cancer) was cultured in DMEM containing 10% FCS. Cell numbers were counted using a Casy cell counting system (Roche Innovatis, Mannheim, Germany).

### RNA Extraction

miRNA purifications from fresh frozen tissue-samples were performed using the miRNeasy Mini Kit (Qiagen). The RNeasy Plus Micro Kit (Qiagen) was used for isolation of RNA from small amounts of cells.

### Quantitative Real-time RT-PCR

For cDNA synthesis the First Strand cDNA Synthesis Kit (Roche Applied Science, Mannheim, Germany) was used and for quantitative RT-PCRs the Power SYBR Green PCR Master Mix (Life Technologies) together with specific primers (Table S1). PPIA (peptidylprolyl isomerase A) was used for normalisation. For miRNA analyses 5 ng total RNA was used for cDNA preparation with TaqMan MicroRNA Reverse Transcription Kit and miRNA-specific primer (Life Technologies). Quantitative RT-PCRs were performed with TaqMan MicroRNA Assays and TaqMan Universal PCR Master Mix (Life Technologies) or miRCURY locked nucleic acid oligonucleotides (LNA) (Exiqon, Vedbæk, Denmark). SnRNA U6 was used for normalisation. For all quantitative RT-PCRs cut off C_T_ values of 35 were used.

### miRNA TaqMan Low Density Array

TLDA Human MicroRNA A Cards v2.0 (Life Technologies) were used to determine miRNA expression profiles. Initially all RNA preparations were tested with a PCR for the reference gene RNU48 for suitability and only samples with a cycle threshold (C_T_) lower than 25 for were further used. For normalisation of the TLDAs, however, snU6 was used because of a more uniform expression pattern and four-fold measurement per array. For all quantitative RT-PCRs cut off C_T_ values of 35 were used.

### Transfection of ES Cell Lines

ES cell lines were transiently transfected with mirVana miRNA mimics (30–100 nM) or Ambion Pre-miR precursors (30–100 nM) (Life Technologies) using the HiPerFect Transfection Reagent (Qiagen). As negative control a miRNA was used which has, as stated by the supplier (Life Technologies), a unique sequence designed such that it does not target any human genes and has been tested in human cell lines and validated to not produce identifiable effects on known miRNA functions. For details to the transfection protocol see Dataset S2.

### Invasion Assay

Cells were precultured in serum-reduced medium (1–3% FCS) for 48 hours and transfected at time-point 0 hours and 24 hours with miR-31 mimics or control. For invasion assays cells were seeded in 24-well BD BioCoat MATRIGEL Invasion Chambers with 8 μm pores in accordance with the manufacture’s protocol (BD Biosciences Pharmingen, San Jose, CA, USA). After cells were transferred into inserts they were transfected a third time. As a chemoattractant, medium containing 10% FCS was used in the lower chamber. After 30–48 hours cells attached to the lower surface of the membrane were fixed, stained (Diff Quik; Polysciences Inc., Warrington, PA, USA) and counted.

### Affymetrix GeneChip Microarray Analysis

TC-71 cells were transfected with miR-31- or negative-mimics in three independent experiments. Total RNA was isolated from cells after 72 hour incubation in serum-reduced medium (1% FCS) and three transfections. Affymetrix GeneChip Human 1.0 ST array (Affymetrix, Santa Clara, CA, USA) runs were performed by the Integrated Functional Genomics (IFG) Unit of the Interdisciplinary Center for Clinical Research (IZKF) at the Medical Faculty of the University of Münster, Germany, and the data were analysed using Genespring GX 12.1 (Agilent Technologies, Santa Clara, CA, USA) with GC-RMA preprocessing of the raw expression data (CEL files). Data were normalized using the RMA (Robust Multi-array Analysis) algorithm.

To determine if the expression of putative miR-31 target genes was altered in ES we used Affymetrix GeneChip Human 1.0 ST arrays to profile a subset of 20 primary ES that had been used for miRNA profiling as above. Transcript summarized data from ES were compared to adult bone marrow MSCs as previously described [13]. All raw cel files were normalized together using gcRMA and transcript summarized in Partek Genomics Suite (Partek, St. Louis, MO, USA).

### Data Deposition

TaqMan Low Density Array raw data and GeneChip Human 1.0 ST array raw data are available at EBI ArrayExpress E-MTAB-1337 and E-MTAB-1334.

### Immunoblotting

Cells were lysed in SDS sample buffer and further analysed as previously described [28]. For detection primary antibodies against VAV3 (#2398; Cell Signaling) and as loading control Actin (C-11) (sc-1615; Santa Cruz Biotechnology, Santa Cruz, CA, USA) were used.

### FACS

For the apoptosis detection at least 5×10^5^ cells were stained with FITC-AnnexinV (BD Biosciences) and propidiumiodide (PI)(AppliChem, Darmstadt, Germany) and analyzed by flow cytometry (BD FACSCalibur, BD Biosciences). For cell cycle analysis at least 5×10^5^ cells were fixed in 80% ethanol overnight at −20°C and stained with PI in PBS containing RNase A and analysed by flow cytometry.

### Statistical Analysis

Analyses of TLDA data were performed with the RealTime StatMiner (Version 4.2, Integromics) software. Unsupervised hierarchical clustering was performed using an unweighted average linkage and Pearson correlation coefficient. For differential gene expression analysis two-tailed student’s t-test was used to calculate p-values. The resulting p-values were further adjusted for multiple comparisons using the Benjamini-Hochberg method to obtain false discovery rate corrected q-values [29].

## Results

### miRNA-expression Profiling of Ewing Sarcoma

**Figure 1:**
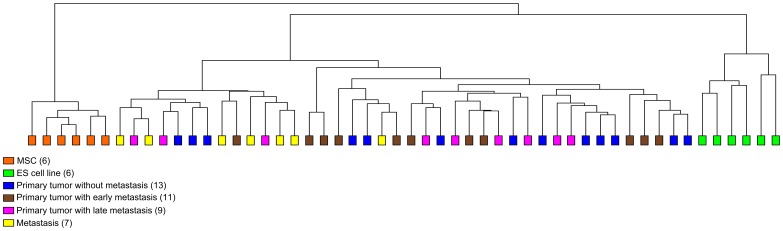
Unsupervised hierarchical clustering of miRNA expression profiles of ES and MSCs. miRNA expression profiles of 52 samples, 6-values were normalized using U6 snRNA to generate relative expression levels. Unsupervised cluster analysis was based on Pearson’s correlation (unweighted average) and performed without any stringent filtering criteria. The samples are separated in two branches, MSC and ES samples and the ES samples are further separated into cell lines and biopsies.

To identify differentially expressed miRNAs in ES TLDAs were used to examine the expression of 377 miRNAs in 40 ES biopsies (13 tumor samples of patients with localised disease and sustained remission for at least five years, 11 primary tumor samples of patients with metastatic disease at presentation or with early systemic relapse within the first year after diagnosis, 9 primary tumor samples of patients with late systemic relapse more than one year after diagnosis, and 7 metastases) [5], 6 ES cell lines (CADO-ES1, RD-ES, RM-82, TC-71, VH-64, WE-68) and 6 *in-vitro* expanded MSC samples of healthy donors. SnRNA U6 was used for normalisation. Unsupervised hierarchical clustering without any filtering criteria divided the samples in two major branches, MSCs and ES samples, with the ES samples further divided into cell lines and biopsies (Figure 1). This indicated a consistently differential miRNA expression in ES samples compared to MSCs, but also consistent differences in miRNA expression in ES cell lines compared to ES biopsies, which were most likely due to the adaption of the ES cell lines to cell culture conditions.

Supervised cluster analyses were then performed to identify miRNAs with aberrant expression in ES by comparing the tumor biopsy samples and the cell lines separately to MSCs as the putative cells of origin of ES (Table S2). Using a 4-fold absolute change and a false discovery rate of 0.05 as cut off, 74 miRNAs were differentially expressed in ES biopsies. Of these 74 miRNAs 35 were also differentially expressed in the comparison of ES cell lines to MSCs (Table S3). For the remaining 28 miRNAs with differential expression in ES biopsies but not in ES cell lines, it could not be excluded that their differential expression in ES biopsies was due to cell culture adaption of the *in-vitro* expanded MSCs used for comparison and these miRNAs were therefore excluded from further analyses. Of the 35 differentially expressed miRNAs 19 were higher and 16 lower expressed in ES samples, with miR-31 showing the highest fold-change value (≈1066-fold; Table 1).

**Table 1:** Differentially expressed miRNAs in ES biopsies compared to MSCs.

		TaqMan Low Density Array analysis			quantitative RT-PCR validation		
	miRNA	Fold Change	p-value	q-value	Fold Change	p-value	q-value
Higher expressed	hsa-miR-126 [18]	209.98	2.82E-27	1.07E-24	404.27	3.02E-12	4.82E-11
	hsa-miR-146b-5p	71.06	1.41E-20	1.39E-18			
	hsa-miR-501-5p	53.94	1.54E-12	4.19E-11	9.53	4.81E-04	5.92E-04
	hsa-miR-598 [41]	31.99	2.84E-08	2.25E-07	31.50	9.45E-07	5.04E-06
	hsa-miR-20b	21.88	1.40E-10	2.05E-09	45.62	1.44E-06	5.75E-06
	hsa-miR-10b	20.10	5.68E-10	6.96E-09	9.11	2.43E-04	3.24E-04
	hsa-miR-491-5p	13.12	2.58E-12	6.53E-11			
	hsa-miR-652 [18], [41]	7.45	1.86E-09	1.97E-08	8.72	2.06E-04	3.22E-04
	hsa-miR-532-5p	6.78	4.60E-10	5.83E-09			
	hsa-miR-340	6.75	2.58E-11	5.16E-10	16.30	2.34E-08	1.87E-07
	hsa-miR-331-5p	6.56	4.06E-05	1.88E-04			
	hsa-miR-422a	6.37	4.72E-08	3.66E-07	20.76	3.61E-06	9.63E-06
	hsa-miR-192	6.06	5.05E-09	4.92E-08			
	hsa-miR-362-5p	6.01	1.54E-09	1.72E-08			
	hsa-miR-128 [18]	5.67	4.31E-05	1.98E-04			
	hsa-miR-342-3p	5.59	5.28E-09	5.01E-08			
	hsa-miR-301a	4.57	2.15E-08	1.74E-07			
	hsa-miR-19b	4.21	4.54E-11	7.50E-10			
	hsa-miR-324-5p	4.17	1.04E-06	6.16E-06			
Lower expressed	hsa-miR-31 [19], [41]	1850.16	1.56E-10	2.19E-09	1065.64	3.25E-06	1.04E-05
	hsa-miR-137	74.67	2.39E-03	6.69E-03			
	hsa-miR-138	70.58	4.47E-06	2.33E-05			
	hsa-miR-431	18.64	7.02E-08	5.23E-07	8.45	1.94E-03	2.22E-03
	hsa-miR-708 [18], [22]	14.37	1.95E-07	1.30E-06	18.35	5.28E-05	9.39E-05
	hsa-miR-100 [19], [20], [41]	14.09	6.94E-08	5.23E-07	14.52	1.64E-05	4.38E-05
	hsa-miR-193a-5p	13.14	1.17E-08	9.87E-08	13.31	3.13E-05	6.26E-05
	hsa-miR-99a	10.49	3.22E-07	2.01E-06	8.53	2.21E-04	3.22E-04
	hsa-miR-221 [20]	6.55	2.11E-05	1.01E-04	2.47	2.21E-02	2.36E-02
	hsa-miR-671-3p	6.06	1.92E-03	5.62E-03			
	hsa-miR-222 [20]	4.97	6.24E-05	2.69E-04			
	hsa-miR-193a-3p [41]	4.94	3.25E-03	8.72E-03			
	hsa-miR-493	4.84	1.61E-04	6.49E-04	2.98	5.56E-02	5.56E-02
	hsa-miR-196b	4.79	2.20E-03	6.20E-03			
	hsa-miR-125b [18]–[20]	4.37	3.12E-03	8.46E-03			
	hsa-miR-376a	4.16	3.27E-03	8.72E-03			

Significant differentially expressed miRNAs with a fold change ≥4 and a q-value <0.05 comparing 40 ES tumor biopsies to 6 MSC samples are shown. We excluded miRNAs that showed differential expression in comparison of ES biopsies to MSCs, but not in comparisons of ES cell lines to MSCs, as it could not be excluded that their differential expression in ES biopsies was due to cell culture adaption of the *in-vitro* expanded MSCs. The q-values were calculated by taking p-values corrected for multiple testing using the Benjamini-Hochberg method. miRNAs whose differential expression has been published in recent studies are marked. For 16 miRNAs differential expression was validated performing TaqMan qRT-PCRs for further 10 ES samples and 4 MSC samples. ES validation samples were also compared to MSCs.

To validate the TLDA data 16 differentially expressed miRNAs were selected for quantitative RT-PCRs using TaqMan-assays with 10 additional ES biopsies, 4 additional *in-vitro* expanded MSC populations and, for further comparisons (see below), also 7 OS biopsies, the most frequent bone tumor in adolescents and young adults. For 14 of the 16 miRNAs differential expression with a q-value less than 0.05 and a fold change ≥4 could be verified (Table 1). As the TLDAs and the validations were performed with the same TaqMan®-assays based on stem-loop PCR [30], the expression of two miRNAs, miR-126 and miR-31 was also analysed using another PCR-method based on LNA-modified oligonucleotides and Sybr-green for quantification (miRCURY LNA miRNA PCR System, Exiqon) and snRNA U6 for normalisation in the 10 additional ES biopsies and 4 additional *in-vitro* expanded MSC populations [31]. Although the extent of differential expression varied significantly between the two PCR based methods (miR-126∶4958 fold upregulated, q = 1.70E-25, with LNA-PCR and 404 fold upregulated with stem-loop PCR; miR-31∶197 fold downregulated, q = 6.94E-11, with LNA-PCR and 1065 fold downregulated with stem-loop PCR) both methods confirmed the strong and statistically significant differential expression of both miRNAs in ES.

The vast majority of ES are caused by two different translocations encoding aberrant fusion transcription factors. To analyse whether the different translocations are associated with different miRNAs expression patterns, 26 primary tumor cases with an EWS-FLI1 translocation were compared to 7 cases with an EWS-ERG translocation performing unsupervised and supervised cluster analysis. The ES samples with the different translocation types were neither separated in the unsupervised cluster analysis (Figure S1A), also when only the miRNAs with the greatest differences in expression with standard deviations higher than 1 or 2 were used, nor were significantly differentially expressed miRNAs (q-value <0.05) identified in the supervised cluster analysis.

To identify miRNAs related to tumor dissemination and prognosis in ES several unsupervised and supervised cluster analyses of primary tumors with different metastatic behaviour and metastases were performed. Unsupervised cluster analyses with various stringencies (all miRNAs or only with miRNAs with standard deviations of >1 or >2) did not separate metastases from all primary tumor samples and did not cluster the primary tumor samples in groups based on absence or presence, time-point and localisation of metastases (Figure S1B and S1C). In supervised comparisons of all primary tumor samples to metastases and between primary tumor samples with differing dissemination time points and with different localisation of metastases no significant differentially expressed miRNAs (FC ≥4, q-value <0.05) that could be validated with further samples were detected.

In summary, 35 miRNAs were identified as differentially expressed in ES compared to MSCs (FC ≥4, q-value <0.05) and no significant differences in miRNA expression were detected between cases with different EWS-translocations and with different dissemination behaviour.

### miR-31 Decreases Proliferation of Several Ewing Sarcoma Cell Lines

**Figure 2:**
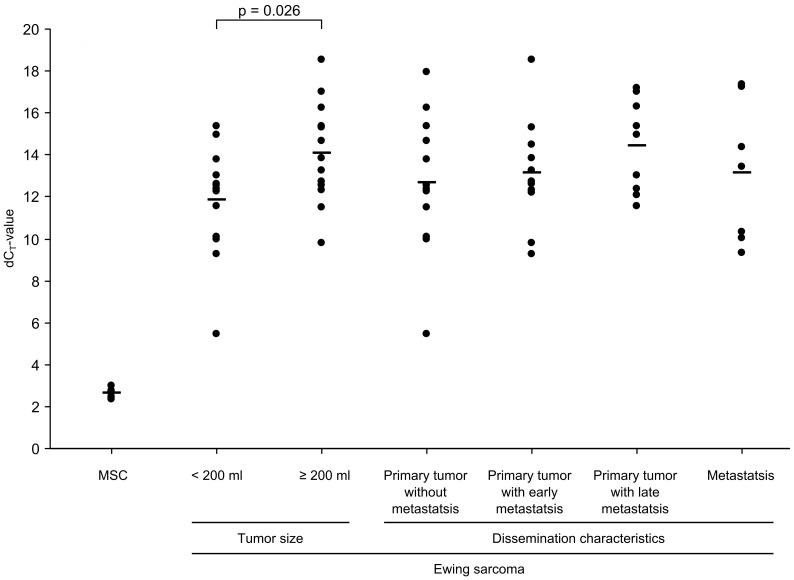
miR-31 expression in mesenchymal stem cells and Ewing sarcomas with different clinicopathological features. miR-31 expression in 6 MSC samples and 40 ES biopsies was determined using TLDAs. Ct-values were normalized using U6 snRNA. The ES samples were dived into different groups based on tumor size (27 primary tumors, for 6 primary tumors this information was not available) or dissemination characteristics.

In this study miR-31 was identified as the most differentially expressed miRNA compared to MSCs (Figure 2). In comparisons of ES to OS miR-31 was 20-fold lower expressed in ES, showing that miR-31 in ES is not only lower expressed in comparison to *in-vitro* expanded MSCs but also in relation to tumor biopsies derived from another bone sarcoma. A comparison of the expression of miR-31 between groups of ES patients with different clinicopathological features (Figure 2) revealed a statistically significant lower miR-31 expression in tumors with a size of >200 ml compared to tumors <200 ml (5-fold reduced expression, p = 0.026).

**Figure 3:**
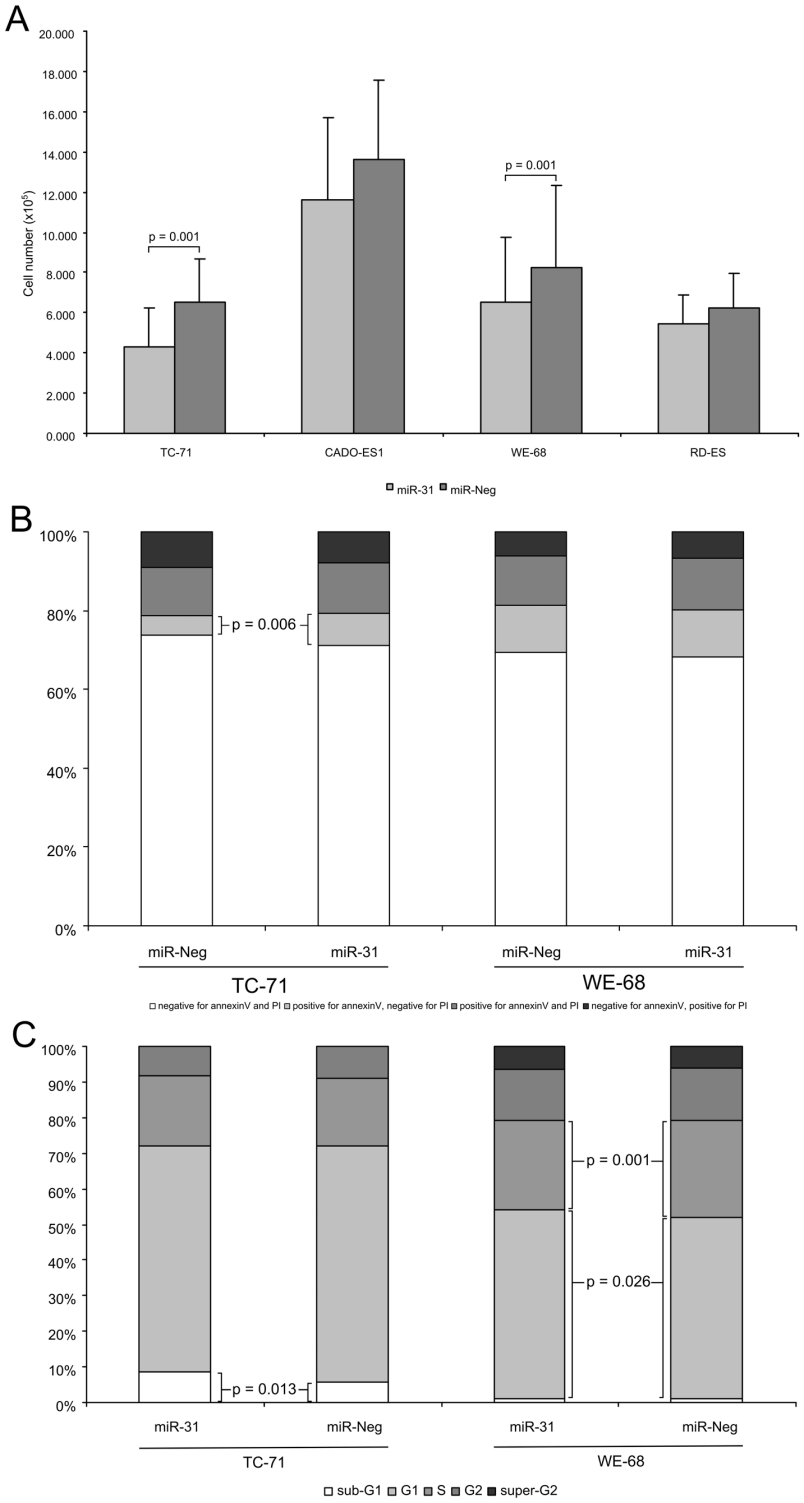
Effects of miR-31 on cell number, apoptosis and proliferation of ES cell lines. (A) miR-31 decreases the proliferation of ES cell lines. Four ES cell lines were transiently transfected with 100 nM miR-31 mimics. After 72 hour miR-31 amounts were comparable to those in MSCs. Shown are mean-values of several independent experiments (TC-71∶18; RD-ES and CADO-ES1∶3; WE-68∶15). (B) Induction of cell death was monitored by FACS analysis of annexinV-FITC and PI stained cells after 72 hours. The percentage of cells stained for annexinV and PI was calculated from 7 for TC-71 and 6 for WE-68 transfections. (C) Effect of miR-31 on proliferation rates. Cells (attached to plate and from culture supernatant) were fixed and stained with PI after 72 h. The percentage of cells in the cell cycle phases were calculated from three different transfections for each cell line. When only cells attached to the plates were analysed in three further transfections the same results were obtained (statistically significant increases in sub-G1 in TC-71 and in G1-phase in WE-68).

To examine the effect of miR-31 on the proliferation of ES, 4 ES cell lines were transiently transfected with miR-31 mimics. With the transfection protocol used the miR-31 amounts achieved in ES cell lines were comparable to those in MSCs (dC_T_s of two MSCs 0.42 and 0.55, TC-71 −0.25, WE-68 −1.09, RD-ES1 −1.37, CADO-ES1 −1.13 (averages from three independent experiments)). After 72 hours cell numbers were reduced in a statistically significant manner in two ES cell lines compared to negative control (TC-71∶67%, p = 0.001; WE-68∶81%, p = 0.001) (Figure 3A) while the slight reduction in two other cell lines was not statistically significant (RD-ES: 88%, p = 0.213; CADO-ES1∶86%, p = 0.368). A similar reduction (TC-71∶60%; WE-68 86%; RD-ES: 90%; CADO-ES: 78%; mean values from 2 experiments) was also observed using pre-miR-31 at an even lower concentration (30 nM)(Figure S2).

The reduced cell numbers could be due to reduced proliferation rates or apoptosis. Therefore, the two cell lines with significant reduced proliferation (TC-71 and WE-68) were analysed for apoptosis by annexinV/PI staining (Figure 3B, Table S4) and for reduced proliferation by PI staining after fixation (Figure 3C, Table S5) with subsequent FACS analysis. For TC-71 both assays indicated a slightly but statistically significant increased fraction of apoptotic cells upon miR-31 transfection (annexinV^+^/PI^−^ fractions in apoptosis assay: 8.15% miR-31, 5.18% miRNA control, p = 0.006; Sub G1 fraction in cell cycle analysis: 8.51% miR-31, 5.68% miRNA control, p = 0.013). No signs of apoptosis could be detected in WE-68 but a small decrease of the fraction of S-phase cells with concomitant increase in the G1-phase cells was observed (G1-phase fraction: 53.47% miR-31, 51,13% miRNA control, p = 0.026; S-phase fraction: 25.29% miR-31, 27.36% miRNA control, p = 0.001).

These results show that miRNA-31 influences proliferation of ES cell lines in ex vivo assays and that the causes for the reduced proliferation, either slightly increased apoptosis or a longer G1-phase, vary between cell lines. Although these differences are small, they could over time accumulate to the differences in cell numbers observed after 72 hour treatment with miR-31. Furthermore, although tumor size does not necessarily reflect proliferation rate, the significantly reduced expression in tumors with sizes >200 ml could be an indication that reduced miR-31 expression contributes also to increased proliferation in primary tumors.

### miR-31 Decreases Invasiveness of Ewing Sarcoma Cell Lines

**Figure 4:**
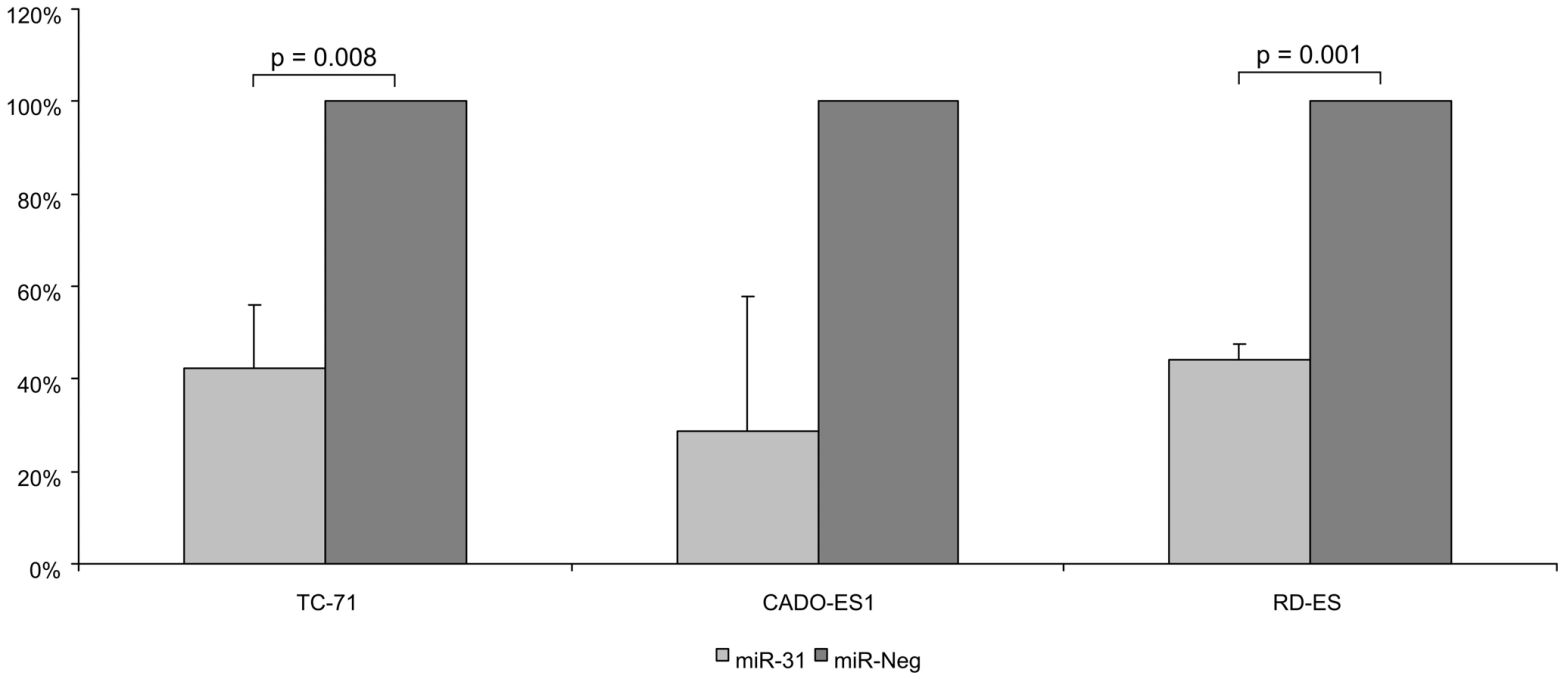
Effects of miR-31 on invasiveness of ES cell lines. Effect of miR-31 on invasion of ES cell lines in ex vivo assays after 72 hours. Invasiveness was analysed in a two chamber cell culture system. After 30 hours or 48 hours cells attached to the membrane in the lower chamber were counted. Shown are average values of three independent experiments. As miR-31 reduced proliferation, cells in additional chambers were treated as the cells used to determine invasion and total cell numbers were counted to calculate a proliferation correction factor with which the numbers of migrated miR-31 transfected cells were multiplied.

miR-31 has been described to play an important role in invasiveness of several cancer types [32]–[36]. However, we observed no correlation between miR-31 expression and metastatic behaviour of primary ES cases (Figure 2). To clarify the role of miR-31 regarding the invasive behaviour of ES, we therefore performed migration and invasion assays with ES cell lines transfected with miR-31 mimics. For all 3 cell lines miR-31 transfected cells showed reduced migration (TC-71∶40%; CADO-ES1∶56%; RD-ES: 26%; cell numbers were corrected for differences in proliferation upon miR-31 transfection)(Figure S3) in two independent experiments and a statistically significant reduced invasion compared to the cells transfected with the negative control (TC-71∶42%, p = 0.008; CADO-ES1∶29%, p = 0.052; RD-ES: 44%, p = 0.001; cell numbers were corrected for differences in proliferation upon miR-31 transfection) in at least three independent experiments (Figure 4).

The significant effects of miR-31 reexpression in ES cell lines in ex vivo assays demonstrate that reduced miR-31 expression is essential for the invasiveness of at least ES cell lines. The lack of a significant different miR-31 expression in primary tumors and metastases or primary tumors with different dissemination characteristics, which however express all very low amounts of miR-31 compared to MSC (Figure 2), together with the ex vivo data, likely indicates that low mir-31 expression is necessary but not sufficient for increased invasiveness and that the low miR-31 expression in non-metastatic ES may contribute in other ways, e.g. by increasing proliferation, to ES pathogenesis.

### miR-31 Targets in Ewing Sarcoma

To directly identify miR-31 targets in ES we generated genome-wide gene expression profiles of TC-71 cells transfected with miR-31 mimics or negative control. Fifteen upregulated and 14 downregulated genes were detected with a 2 fold change cut-off (q-value <0.05)(Table 2). To evaluate which of the downregulated targets may be directly modulated by miR-31, we employed the target prediction algorithm DIANA microT v4.0. STK40 was the only one with seed matches for miR-31.

**Table 2:** mRNAs differentially expressed in miR-31 transfected TC-71 cell line compared to negative control.

	Gene symbol	FC	p-value	q-value
Downregulated	RBMS1	2.84	2.60E-04	1.40E-02
	SERTAD4	2.76	5.52E-05	9.10E-03
	TMEM109	2.45	5.96E-05	9.27E-03
	TP53INP1	2.42	1.17E-03	2.52E-02
	RHOBTB3	2.37	7.10E-05	9.77E-03
	PRPH	2.24	2.26E-05	6.92E-03
	PDGFRB	2.21	4.61E-06	4.44E-03
	STK40	2.19	4.08E-06	4.20E-03
	SLCO4C1	2.17	2.57E-05	6.92E-03
	VGLL3	2.08	2.25E-06	3.09E-03
	SLC16A2	2.08	1.24E-04	1.17E-02
	PCBD1	2.04	1.37E-04	1.21E-02
	TXNIP	2.04	2.23E-04	1.34E-02
	NTS	2.02	1.24E-04	1.17E-02
Upregulated	FRG2	5.41	1.10E-06	2.71E-03
	ETV5	2.73	1.39E-05	6.11E-03
	ESRP1	2.62	3.59E-04	1.57E-02
	ZC3H6	2.60	2.76E-07	1.99E-03
	SEMA3C	2.55	2.85E-05	6.98E-03
	ACTA2	2.51	4.77E-05	8.65E-03
	PPIL6	2.48	5.63E-05	9.10E-03
	DUSP6	2.44	4.49E-05	8.33E-03
	SPRY4	2.32	3.80E-04	1.62E-02
	CCDC113	2.13	4.77E-04	1.72E-02
	CCND3	2.08	1.24E-04	1.17E-02
	DDIT3	2.07	9.16E-04	2.25E-02
	MT1F	2.04	4.52E-04	1.69E-02
	C1D	2.03	2.79E-05	6.92E-03
	WSB1	2.02	7.86E-04	2.12E-02

TC-71 was transfected three times with 100 nM miR-31 or negative control mimics and genome-wide mRNA expression was quantified using Affymetrix GeneChip Human 1.0 ST arrays. Mean values of three independent experiments were used for detection of differentially expressed mRNAs. The 29 genes with a FC >2 and a q-value <0.05 are displayed.

Next, we used indirect methods to identify other potential direct miR-31 targets in ES. Using three target prediction platforms (DIANA microT v4.0, PicTar, TargetScan5) we identified, in addition to the already known ITGA5, RDX and RhoA [35], 15 genes as potential miR-31 targets given their detection by all three algorithms (*CACNB2, CAMK2D, FZD3, NFAT5, NR5A2, NUMB, PPP3CA, RASA1, SEPHS1, SH2D1A, SLC1A2, STX12, VAPB, VAV3, YWHAE*). Genome-wide mRNA expression profiles of 20 ES biopsies and three MSC samples were then examined for differential expression of these 19 putative direct miR-31 target genes (*STK40, ITGA5, RDX, RHOA*, plus the 15 genes identified by target prediction algorithms). Ten of the 19 genes were differentially expressed in ES compared to MSCs (q-value <0.05) and four of these genes showed a significant higher expression in the ES biopsies (q-value <0.05 and median fold change >2): CACNB2 33.3 fold, FZD3 6.4 fold, RDX 2.1 fold and VAV3 2.5 fold (Figure S4A). However, attempts to validate CACNB2 and VAV3 as miR-31 targets experimentally were unsuccessful (Figures S4B–E).

## Discussion

In comparisons of the expression patterns of 377 miRNAs in ES biopsies and cell lines with MSCs as the putative cellular origin of ES [10]–[12], 35 differentially expressed miRNAs were identified. Ten of these were also described in other recent studies of ES, which were predominantly focussed on the identification of EWS/FLI regulated miRNAs. For most of the 16 lower but only a minor fraction of the 19 higher expressed miRNAs aberrant expression has been described in other malignancies.

In addition to the identification of miRNAs differentially expressed in all ES samples, we also examined an association of miRNAs with specific features of subgroups of ES such as translocation type or dissemination characteristics. However, no differences in miRNA expression related to this features were identified at high stringency (fold change >4 and p<0.05). Here, at first, limitations regarding group sizes and that only 377 of the more than 1500 miRNAs were analysed has to be considered. Furthermore, imprecise grouping regarding the dissemination characteristics may hamper identification of relevant miRNAs, as in up to 50% of the patients with a diagnosis of localised disease by conventional means micro-metastasis were already detectable by RT-PCR, although this has so far not been confirmed by other studies [37]. These limitations may be the cause that in our study miR-34, which has recently been shown to be significantly upregulated in patients without any adverse events [24], was only 2.23-fold higher expressed (p = 0.002) in primary tumors from patients without metastasis compared to tumors from patients who had metastases at presentation or within the first year of diagnosis. However, even when these limitations of our study are taken into account, the comparisons between various groups of ES indicate that the expression pattern of the 377 miRNAs analysed is relatively uniform in ES samples. Regarding the different translocation types this is in line with a lack of differential mRNA expression and indistinguishable clinical behaviour [38], [39]. This consistent miRNA expression pattern could be due to the uniform presence of an aberrant fusion transcription factor in all ES cases as the dominant determinator of the tumor cell phenotype, regulating the expression of more than 1000 genes [9], [40], and likely also the expression of many miRNAs. However, as in other tumor types, where different transforming events may cause a greater variability in miRNA expression, also in ES additional genomic aberrations may influence miRNA expression, and this could be, in addition to the usage of different detection platforms, the cause for the small overlap with EWSR1/FLI1 regulated miRNAs identified in other studies.

Although miR-31 was the lowest expressed miRNA in ES in comparison to MSCs in this study, low expression of miR-31 was only noticed in two of the other studies on miRNAs in ES but not further examined [19], [41]. The lack of detection of differential miR-31 expression in most other studies on miRNAs in ES could be related to the causes for the reduced miR-31 expression. Although EWS/FLI may influence miR-31 expression via its direct target EZH2 [42], other factors likely also influence miR-31 expression and hamper the detection of differential miR-31 expression in cell lines with or without EWS/FLI expression. One factor could be deletions encompassing the *MIR31* gene, which have been detected in 4 of 11 ES cell lines (among them TC-71 and RD-ES) and in 3 of 26 primary ES and most likely contribute in a significant fraction of cases to the lack or reduced expression of miR-31 (Savola et al 2007; Cancer Cell Line Encyclopedia of the Broad Institute).

Reduced miR-31 expression has also been observed in many other cancer types and been associated with increased proliferation, migration and invasion of tumor cells [32], [34], [36], [43], [44] and the degree of reduced miR-31 expression correlated with the metastatic potential of human breast cancer cell lines [45]. A direct comparison of miR-31 expression in ES, breast cancer and OS cell lines by quantitative RT-PCRs (Table S6) revealed that in 2 ES cell lines miR-31 expression was even lower than in metastatic breast cancer and between 17 and 7383 fold lower than in OS cell lines. Indeed, transfection of ES cell lines with a miR-31 mimic led to decreased proliferation and invasion in ex vivo assays. When miR-31 expression was related to clinicopathological features of ES biopsies, a significantly lower expression of miR-31 was detected in tumors with larger sizes. Together, these results identify miR-31 as a potential tumor suppressor in ES whose influence on proliferation and invasiveness should be further investigated in in vivo models. Interestingly, beside miR-31 several other miRNAs (miR-10b, miR-100, miR-138 and miR-196), whose expression levels have been related to the dissemination characteristics of various tumor types [46], were differentially expressed in comparisons of all ES to MSCs but not between primary ES cases and metastases. This also points to a uniform miRNA expression pattern in ES compared to other tumor types and may indicate that ES have a uniformly high propensity for dissemination.

In summary, we identify 35 miRNAs consistently differentially expressed in ES biopsies and cell lines compared to MSCs, and demonstrate that the lower expression of miR-31, the miRNA with the strongest differential expression in ES, contributes to the proliferation and invasiveness of ES cell lines in ex vivo assays.

## Supporting Information

Figure S1 Unsupervised hierarchical clustering of miRNA expression profiles for the 33 primary tumor biopsy samples. An unweighted average method and a Pearson correlation similarity measure were used. Three different designations of the tumors are displayed. In (A) the translocation type of the samples, in (B) the absence or presence of metastasis and time-point of metastasis and in (C) the assignment to clinical risk groups is shown. In the primary cases without metastasis only samples of patients with an at least five year event-free survival were included. Patients with localized disease at diagnosis were assigned to the low risk group, patients who had pulmonary metastases to the middle risk group and patients with extrapulmonary metastases to the high risk group, as presence and localisation of metastases is an important prognostic factor with three-year survival rates of 75%, 45–50% and 25% for patients without, with pulmonary and with bone metastasis, respectively [1], [2]. References: 1. Ladenstein R, Potschger U, Le Deley MC, Whelan J, Paulussen M, et al. (2010) Primary disseminated multifocal ewing sarcoma: Results of the euro-EWING 99 trial. J Clin Oncol 28∶3284–3291. 2. Haeusler J, Ranft A, Boelling T, Gosheger G, Braun-Munzinger G, et al. (2010) The value of local treatment in patients with primary, disseminated, multifocal ewing sarcoma (PDMES). Cancer 116∶443–450.
(TIF)

Figure S2 Pre-miR31 decreases the proliferation of Ewing sarcoma cell lines. Four ES cell lines were transiently transfected with 30 nM pre-miR-31 or the corresponding negative control from the same supplier (Ambion). Cell numbers were determined after 72 hours. Shown are mean-values of two independent experiments.
(TIF)

Figure S3 miR-31 decreases migration of Ewing sarcoma cell lines. Effect of miR-31 on migration of ES cell lines in ex vivo assays after 72 hours. Migration was analysed in a two chamber cell culture system. After 30 hours cells attached to the membrane in the lower chamber were counted. Shown are average values of two (TC-71 and RD-ES) or three (CADO-ES1) independent experiments. As miR-31 reduced proliferation, cells in additional chambers were treated as the cells used to determine migration and total cell numbers were counted to calculate a proliferation correction factor with which the numbers of migrated miR-31 transfected cells were multiplied.
(TIF)

Figure S4 Identification and validation of miR-31 targets in ES. (A) Expression of 19 potential miR-31 targets in 20 ES and 3 MSC samples was examined. Shown are the four genes for which significant higher expression (q-value <0.05, fold change >2) in ES compared to MSCs was observed. (B) To detect the effects of miR-31 on CACNB2 and VAV3 transcripts quantitative RT-PCRs were performed after 72 hours and transfection of TC-71 with 100 nM miR-31 mimics or negative-control. Shown are mean-values of six independent experiments. At the transcript level CACNB2 and VAV3 showed a small but statistically significant downregulation in TC-71 following expression of the miR-31 mimic (CACNB2: FC 1.56, p = 0.001; VAV3: FC 1.72, p = 0.032), but no significant changes were observed in three other ES cell lines (not shown) (C) Changes in the expression of CACNB2 and VAV3 were also analysed at the protein level. Downregulation of VAV3 protein was observed in three of four ES cell lines following miR-31 mimic transfection. CACNB2 did not show a reduction in any cell line (not shown). (D) Potential miR-31 binding sites in the VAV3 3′-untranslated region. The graph is adapted from target prediction software DIANA microT v4.0. Mutations introduced into the seed region are marked in yellow (in both instances C>G). (E) To analyse whether VAV3 is a direct miR-31 target, the two potential miR-31 binding sites from the 3′-untranslated region of VAV3 were cloned together in a luciferase reporter plasmid (Promega, Madison, WI, USA). As negative control a single nucleotide exchange was introduced in each seed region. Luciferase reporter plasmids with the two potential miR-31 binding sites from VAV3 and controls with mutations in the seed regions were cotransfected with miR-31, negative control or miR-31 inhibitor into HEK293T cells (1×10^5^ HEK293T cells, pGL reporter (1 μg) together with pRL (10 ng) and miR-31-mimics or control miRNA or miR-31 inhibitor (mirVana; Life Technologies), each 200 nM, using the FuGene reagent (Promega). After 24 h Luciferase activity was determined with the Dual-Glo Luciferase Assay System in a TD-20/20 luminometer (Promega)). For each transfection firefly luciferase activity was normalised to renilla luciferase activity (devision of both values) and then the normalised value for the wildtype reporter divided through the normalised value of the mutant reporter. Also in direct comparisons of cotransfection of miR-31, control miRNA or miR-31 inhibitor with the wildtype reporter no differences in reporter activity were observed.
(TIF)

Table S1 Primer sequences for qRT-PCR and cloning of the potential VAV3 miR-31 binding sites.
(DOCX)

Table S2 miRNAs differentially expressed comparing miRNA expression profiles generated with TLDAs of ES-biopsies to MSCs.
(DOCX)

Table S3 miRNAs differentially expressed comparing miRNA expression profiles generated with TLDAs of ES cell lines to MSCs.
(DOCX)

Table S4 Values from the apoptosis assays corresponding to Figure 2B.
(DOCX)

Table S5 Values from the cell cycle analysis corresponding to Figure 2C.
(DOCX)

Table S6 Expression of miR-31 in MSCs and different cancer cell lines (ES, OS, breast cancer).
(DOCX)

Dataset S1 Characterisation of mesenchymal stem cells.
(DOC)

Dataset S2 Establishment of the transfection protocol.
(DOC)
