# Supplementary figures and images for: Differentially Expressed miRNAs in Ewing Sarcoma Compared to Mesenchymal Stem Cells: Low miR-31 Expression with Effects on Proliferation and Invasion
Source: PLoS One. 2014 Mar 25;9(3):e93067. doi: 10.1371/journal.pone.0093067 (PMC3965523; doi:10.1371/journal.pone.0093067)

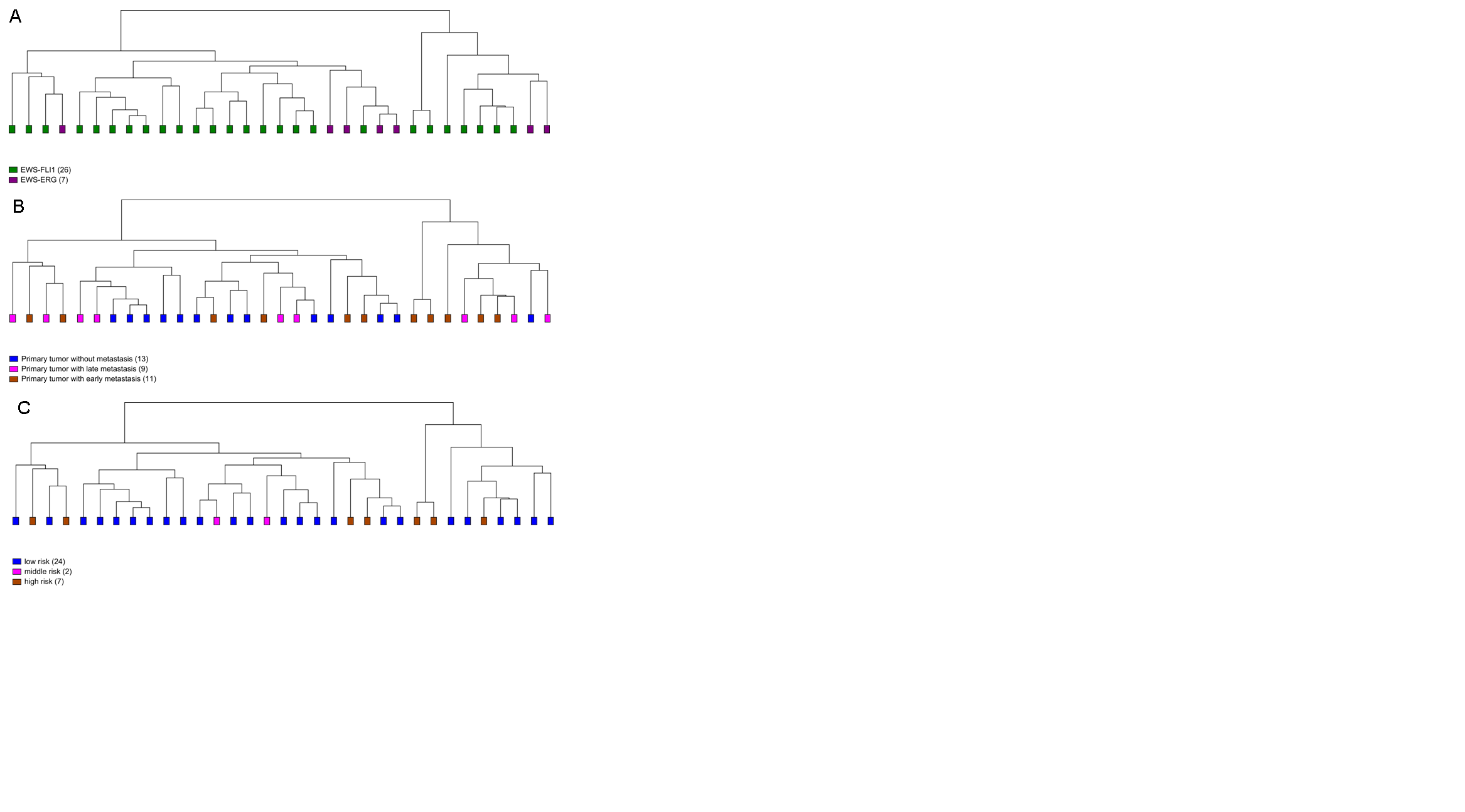

Supplement: Figure S1 — Unsupervised hierarchical clustering of miRNA expression profiles for the 33 primary tumor biopsy samples. An unweighted average method and a Pearson correlation similarity measure were used. Three different designations of the tumors are displayed. In (A) the translocation type of the samples, in (B) the absence or presence of metastasis and time-point of metastasis and in (C) the assignment to clinical risk groups is shown. In the primary cases without metastasis only samples of patients with an at least five year event-free survival were included. Patients with localized disease at diagnosis were assigned to the low risk group, patients who had pulmonary metastases to the middle risk group and patients with extrapulmonary metastases to the high risk group, as presence and localisation of metastases is an important prognostic factor with three-year survival rates of 75%, 45–50% and 25% for patients without, with pulmonary and with bone metastasis, respectively [1], [2]. References: 1. Ladenstein R, Potschger U, Le Deley MC, Whelan J, Paulussen M, et al. (2010) Primary disseminated multifocal ewing sarcoma: Results of the euro-EWING 99 trial. J Clin Oncol 28∶3284–3291. 2. Haeusler J, Ranft A, Boelling T, Gosheger G, Braun-Munzinger G, et al. (2010) The value of local treatment in patients with primary, disseminated, multifocal ewing sarcoma (PDMES). Cancer 116∶443–450. (TIF) [file pone.0093067.s001.tif]

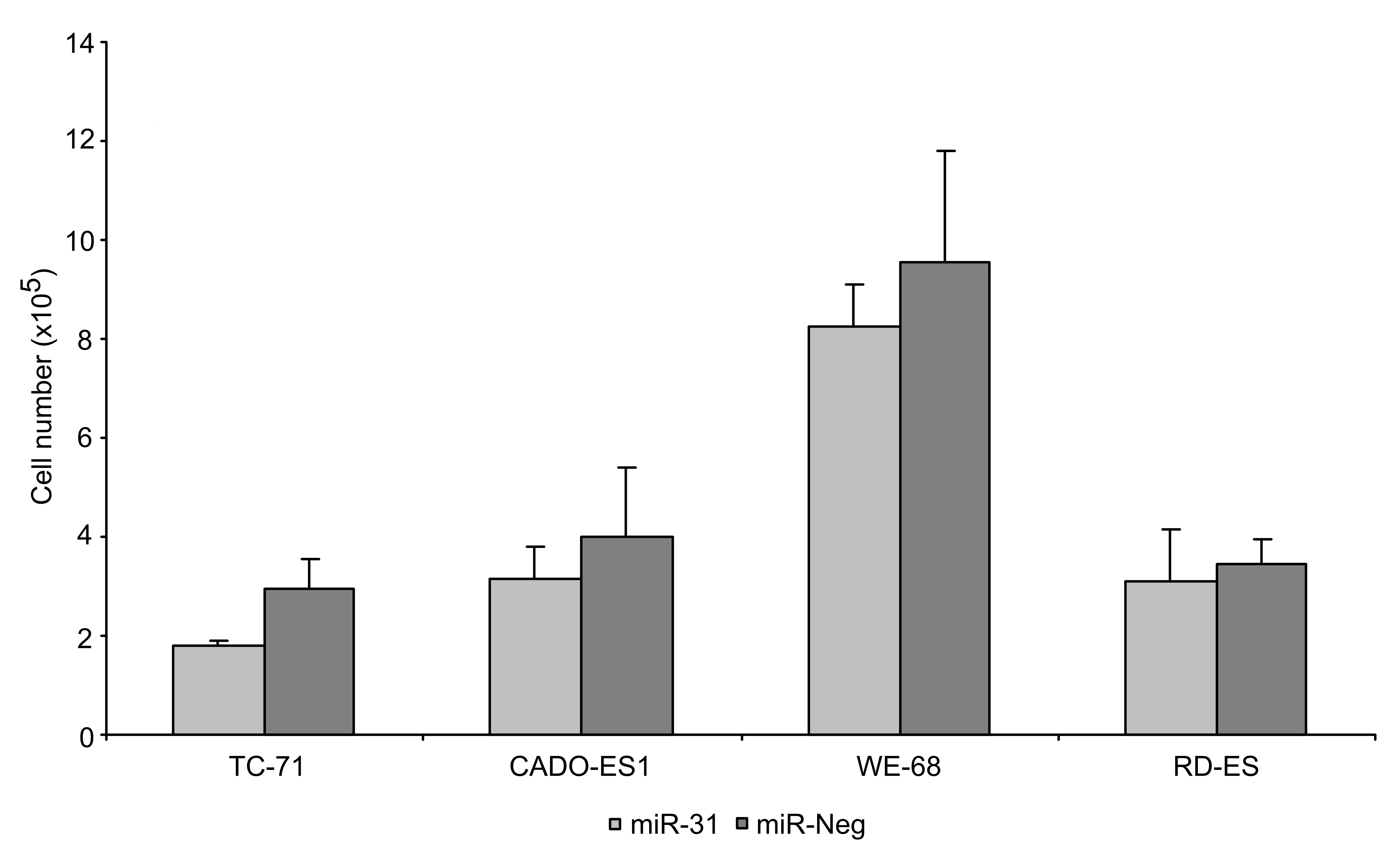

Supplement: Figure S2 — Pre-miR31 decreases the proliferation of Ewing sarcoma cell lines. Four ES cell lines were transiently transfected with 30 nM pre-miR-31 or the corresponding negative control from the same supplier (Ambion). Cell numbers were determined after 72 hours. Shown are mean-values of two independent experiments. (TIF) [file pone.0093067.s002.tif]

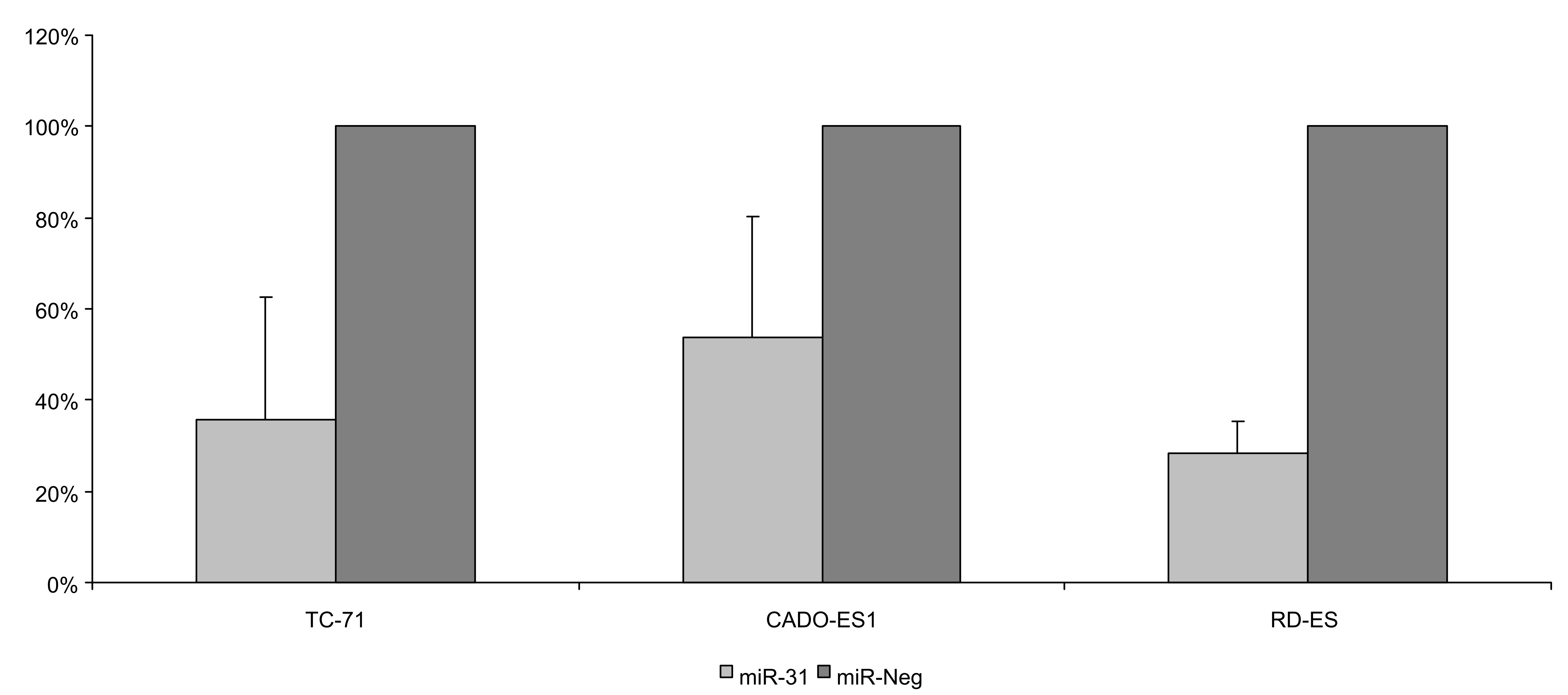

Supplement: Figure S3 — miR-31 decreases migration of Ewing sarcoma cell lines. Effect of miR-31 on migration of ES cell lines in ex vivo assays after 72 hours. Migration was analysed in a two chamber cell culture system. After 30 hours cells attached to the membrane in the lower chamber were counted. Shown are average values of two (TC-71 and RD-ES) or three (CADO-ES1) independent experiments. As miR-31 reduced proliferation, cells in additional chambers were treated as the cells used to determine migration and total cell numbers were counted to calculate a proliferation correction factor with which the numbers of migrated miR-31 transfected cells were multiplied. (TIF) [file pone.0093067.s003.tif]

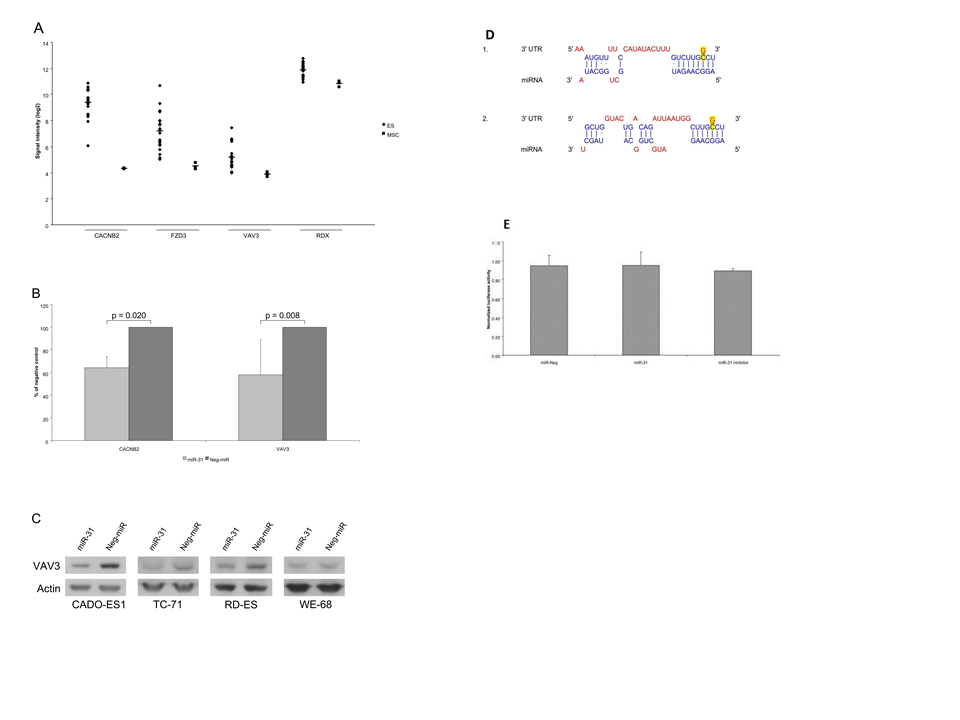

Supplement: Figure S4 — Identification and validation of miR-31 targets in ES. (A) Expression of 19 potential miR-31 targets in 20 ES and 3 MSC samples was examined. Shown are the four genes for which significant higher expression (q-value <0.05, fold change >2) in ES compared to MSCs was observed. (B) To detect the effects of miR-31 on CACNB2 and VAV3 transcripts quantitative RT-PCRs were performed after 72 hours and transfection of TC-71 with 100 nM miR-31 mimics or negative-control. Shown are mean-values of six independent experiments. At the transcript level CACNB2 and VAV3 showed a small but statistically significant downregulation in TC-71 following expression of the miR-31 mimic (CACNB2: FC 1.56, p = 0.001; VAV3: FC 1.72, p = 0.032), but no significant changes were observed in three other ES cell lines (not shown) (C) Changes in the expression of CACNB2 and VAV3 were also analysed at the protein level. Downregulation of VAV3 protein was observed in three of four ES cell lines following miR-31 mimic transfection. CACNB2 did not show a reduction in any cell line (not shown). (D) Potential miR-31 binding sites in the VAV3 3′-untranslated region. The graph is adapted from target prediction software DIANA microT v4.0. Mutations introduced into the seed region are marked in yellow (in both instances C>G). (E) To analyse whether VAV3 is a direct miR-31 target, the two potential miR-31 binding sites from the 3′-untranslated region of VAV3 were cloned together in a luciferase reporter plasmid (Promega, Madison, WI, USA). As negative control a single nucleotide exchange was introduced in each seed region. Luciferase reporter plasmids with the two potential miR-31 binding sites from VAV3 and controls with mutations in the seed regions were cotransfected with miR-31, negative control or miR-31 inhibitor into HEK293T cells (1×10^5^ HEK293T cells, pGL reporter (1 μg) together with pRL (10 ng) and miR-31-mimics or control miRNA or miR-31 inhibitor (mirVana; Life Technologies), each 200 nM, using the [file pone.0093067.s004.tif]
