# Supplementary material for: Differentially Expressed miRNAs in Ewing Sarcoma Compared to Mesenchymal Stem Cells: Low miR-31 Expression with Effects on Proliferation and Invasion
Source: PLoS One. 2014 Mar 25;9(3):e93067. doi: 10.1371/journal.pone.0093067 (PMC3965523; doi:10.1371/journal.pone.0093067)
Supplement: Table S1 — Primer sequences for qRT-PCR and cloning of the potential VAV3 miR-31 binding sites. (DOCX) [file pone.0093067.s005.docx]

**Table S1.** Primer sequences for qRT-PCR and cloning of the potential VAV3 miR-31 binding sites.

| Primer name | Primer sequence (5' → 3') |
| --- | --- |
| CACNB2-US2 | CCTCAACGTCCAGATGGTAGCAGC |
| CACNB2-LS1 | CCTCCAGATAGTCGGCAAGGTGC |
| IGTA5-US1 | CAGCTATGGCGTCCCACTGTGG |
| IGTA5-LS2 | CGGTGCCATATGGGAGGGAGC |
| PPIA-12a | TGGACCCAACACAAATGGTTCC |
| PPIA-12b | CATGGCCTCCACAATATTCATGC |
| VAV3-US3 | CAACTCTGCAGTTTCCATACAAGGAGC |
| VAV3-LS4 | CACAGAAGTCATACCGAGCGATGG |
| VAV3-1-Xba-US | CTGCATTTCTGGCTGTTCAACATCC |
| VAV3-1-WT-LS | GCTGTTTCTTGCACAGCTCTAGGCAAG |
| VAV3-1--M-LS | GCTGTTTCTTGCACAGCTCTAGCCAAG |
| VAV3-2-US | GGGTTTCCTAGCAGAGGATATTGGAGC |
| VAV3-2-WT-LS | CGAGGGCTGCATACAGGCAAGAC |
| VAV3-2-M-LS | CGAGGGCTGCATACAGCCAAGAC |
