# Supplementary material for: Differentially Expressed miRNAs in Ewing Sarcoma Compared to Mesenchymal Stem Cells: Low miR-31 Expression with Effects on Proliferation and Invasion
Source: PLoS One. 2014 Mar 25;9(3):e93067. doi: 10.1371/journal.pone.0093067 (PMC3965523; doi:10.1371/journal.pone.0093067)
Supplement: Table S2 — miRNAs differentially expressed comparing miRNA expression profiles generated with TLDAs of ES-biopsies to MSCs. (DOCX) [file pone.0093067.s006.docx]

**Table S2.** miRNAs differentially expressed comparing miRNA expression profiles generated with TLDAs of ES-biopsies to MSCs.

|  | miRNA | FC | p-value | q-value |
| --- | --- | --- | --- | --- |
|  |  |  |  |  |
| Higher expressed | hsa-miR-95 | 323.45 | 3.68E-15 | 1.40E-13 |
|  | hsa-miR-223 | 270.23 | 1.13E-12 | 3.30E-11 |
|  | hsa-miR-126 | 209.98 | 2.82E-27 | 1.07E-24 |
|  | hsa-miR-486-5p | 101.72 | 1.63E-09 | 1.77E-08 |
|  | hsa-miR-142-3p | 99.51 | 6.68E-15 | 2.31E-13 |
|  | hsa-miR-139-5p | 87.38 | 6.97E-16 | 3.79E-14 |
|  | hsa-miR-133a | 73.63 | 7.63E-05 | 3.26E-04 |
|  | hsa-miR-146b-5p | 71.06 | 1.41E-20 | 1.39E-18 |
|  | hsa-miR-501-5p | 53.94 | 1.54E-12 | 4.19E-11 |
|  | hsa-miR-483-5p | 43.02 | 3.25E-11 | 5.88E-10 |
|  | hsa-miR-598 | 31.99 | 2.84E-08 | 2.25E-07 |
|  | hsa-miR-362-3p | 28.85 | 2.97E-09 | 3.05E-08 |
|  | hsa-miR-195 | 27.43 | 3.60E-15 | 1.40E-13 |
|  | hsa-miR-194 | 25.30 | 3.48E-09 | 3.48E-08 |
|  | hsa-miR-20b | 21.88 | 1.40E-10 | 2.05E-09 |
|  | hsa-miR-10b | 20.10 | 5.68E-10 | 6.96E-09 |
|  | hsa-miR-1 | 18.48 | 1.57E-02 | 3.43E-02 |
|  | hsa-miR-200a | 17.60 | 8.73E-04 | 2.73E-03 |
|  | hsa-miR-181a | 17.25 | 4.39E-11 | 7.50E-10 |
|  | hsa-miR-502-5p | 14.79 | 2.80E-06 | 1.54E-05 |
|  | hsa-miR-491-5p | 13.12 | 2.58E-12 | 6.53E-11 |
|  | hsa-miR-101 | 11.73 | 5.65E-12 | 1.26E-10 |
|  | hsa-miR-204 | 11.39 | 2.48E-04 | 9.72E-04 |
|  | hsa-miR-203 | 9.36 | 4.22E-04 | 1.54E-03 |
|  | hsa-miR-190 | 9.09 | 4.07E-04 | 1.50E-03 |
|  | hsa-miR-124 | 8.99 | 4.60E-03 | 1.20E-02 |
|  | hsa-miR-328 | 8.54 | 3.41E-06 | 1.82E-05 |
|  | hsa-miR-107 | 8.40 | 1.05E-03 | 3.23E-03 |
|  | hsa-miR-660 | 7.82 | 3.25E-10 | 4.26E-09 |
|  | hsa-miR-652 | 7.45 | 1.86E-09 | 1.97E-08 |
|  | hsa-miR-202 | 7.32 | 5.16E-03 | 1.32E-02 |
|  | hsa-miR-210 | 7.16 | 5.79E-06 | 2.97E-05 |
|  | hsa-miR-489 | 7.15 | 3.62E-04 | 1.35E-03 |
|  | hsa-miR-26b | 6.97 | 2.72E-11 | 5.17E-10 |
|  | hsa-miR-146a | 6.95 | 2.08E-08 | 1.72E-07 |
|  | hsa-miR-532-5p | 6.78 | 4.60E-10 | 5.83E-09 |
|  | hsa-miR-340 | 6.75 | 2.58E-11 | 5.16E-10 |
|  | hsa-miR-331-5p | 6.56 | 4.06E-05 | 1.88E-04 |
|  | hsa-miR-500 | 6.55 | 5.77E-09 | 5.34E-08 |
|  | hsa-miR-422a | 6.37 | 4.72E-08 | 3.66E-07 |
|  | hsa-miR-215 | 6.18 | 1.52E-02 | 3.33E-02 |
|  | hsa-miR-423-5p | 6.18 | 8.31E-05 | 3.51E-04 |
|  | hsa-miR-192 | 6.06 | 5.05E-09 | 4.92E-08 |
|  | hsa-miR-362-5p | 6.01 | 1.54E-09 | 1.72E-08 |
|  | hsa-miR-502-3p | 5.70 | 8.45E-06 | 4.17E-05 |
|  | hsa-miR-128 | 5.67 | 4.31E-05 | 1.98E-04 |
|  | hsa-miR-342-3p | 5.59 | 5.28E-09 | 5.01E-08 |
|  | hsa-miR-30b | 5.57 | 6.04E-11 | 9.18E-10 |
|  | hsa-miR-885-5p | 5.57 | 9.52E-03 | 2.28E-02 |
|  | hsa-miR-505 | 5.31 | 1.38E-02 | 3.09E-02 |
|  | hsa-miR-542-3p | 5.25 | 2.28E-02 | 4.73E-02 |
|  | hsa-miR-26a | 4.85 | 2.48E-10 | 3.37E-09 |
|  | hsa-miR-301a | 4.57 | 2.15E-08 | 1.74E-07 |
|  | hsa-miR-32 | 4.57 | 1.76E-06 | 9.99E-06 |
|  | hsa-miR-532-3p | 4.29 | 1.24E-06 | 7.22E-06 |
|  | hsa-miR-132 | 4.27 | 2.30E-06 | 1.29E-05 |
|  | hsa-miR-19b | 4.21 | 4.54E-11 | 7.50E-10 |
|  | hsa-miR-324-5p | 4.17 | 1.04E-06 | 6.16E-06 |
|  | hsa-miR-148a | 3.97 | 1.88E-04 | 7.45E-04 |
|  | hsa-let-7c | 3.87 | 6.21E-04 | 2.05E-03 |
|  | hsa-miR-20a | 3.79 | 1.73E-07 | 1.20E-06 |
|  | hsa-miR-425 | 3.76 | 2.65E-07 | 1.71E-06 |
|  | hsa-miR-19a | 3.73 | 6.25E-09 | 5.66E-08 |
|  | hsa-miR-374b | 3.65 | 1.85E-07 | 1.26E-06 |
|  | hsa-miR-92a | 3.48 | 4.10E-06 | 2.17E-05 |
|  | hsa-miR-30c | 3.47 | 1.10E-07 | 7.88E-07 |
|  | hsa-miR-200c | 3.47 | 3.69E-03 | 9.75E-03 |
|  | hsa-miR-106b | 3.47 | 3.16E-07 | 2.00E-06 |
|  | hsa-miR-106a | 3.45 | 9.23E-08 | 6.75E-07 |
|  | hsa-miR-17 | 3.36 | 1.70E-07 | 1.20E-06 |
|  | hsa-miR-296-5p | 3.18 | 1.27E-02 | 2.88E-02 |
|  | hsa-miR-324-3p | 3.17 | 1.54E-06 | 8.88E-06 |
|  | hsa-miR-186 | 3.16 | 7.39E-09 | 6.53E-08 |
|  | hsa-let-7g | 3.09 | 2.64E-07 | 1.71E-06 |
|  | hsa-miR-301b | 3.03 | 5.37E-04 | 1.94E-03 |
|  | hsa-miR-16 | 3.02 | 1.03E-06 | 6.16E-06 |
|  | hsa-miR-361-5p | 2.94 | 1.11E-04 | 4.62E-04 |
|  | hsa-miR-23a | 2.78 | 3.28E-03 | 8.72E-03 |
|  | hsa-miR-130a | 2.75 | 5.46E-04 | 1.96E-03 |
|  | hsa-miR-199a-5p | 2.74 | 2.90E-03 | 7.91E-03 |
|  | hsa-miR-103 | 2.70 | 1.29E-04 | 5.29E-04 |
|  | hsa-miR-374a | 2.61 | 6.17E-06 | 3.09E-05 |
|  | RNU48 | 2.58 | 1.79E-04 | 7.16E-04 |
|  | hsa-miR-212 | 2.46 | 5.36E-03 | 1.37E-02 |
|  | hsa-miR-345 | 2.45 | 1.25E-04 | 5.14E-04 |
|  | hsa-miR-331-3p | 2.38 | 4.78E-05 | 2.14E-04 |
|  | hsa-miR-320 | 2.22 | 2.08E-03 | 6.01E-03 |
|  | hsa-miR-140-3p | 2.12 | 5.45E-03 | 1.38E-02 |
|  | hsa-miR-185 | 2.10 | 2.09E-02 | 4.39E-02 |
|  | hsa-miR-148b | 2.08 | 1.15E-03 | 3.44E-03 |
|  | hsa-miR-191 | 2.05 | 5.91E-05 | 2.58E-04 |
|  | hsa-let-7d | 1.60 | 1.45E-02 | 3.22E-02 |
|  | hsa-miR-590-5p | 1.53 | 8.22E-03 | 2.00E-02 |
|  |  |  |  |  |
| Lower expressed | hsa-miR-31 | 1850.16 | 1.56E-10 | 2.19E-09 |
|  | hsa-miR-137 | 74.67 | 2.39E-03 | 6.69E-03 |
|  | hsa-miR-138 | 70.58 | 4.47E-06 | 2.33E-05 |
|  | hsa-miR-431 | 18.64 | 7.02E-08 | 5.23E-07 |
|  | hsa-miR-708 | 14.37 | 1.95E-07 | 1.30E-06 |
|  | hsa-miR-100 | 14.09 | 6.94E-08 | 5.23E-07 |
|  | hsa-miR-193a-5p | 13.14 | 1.17E-08 | 9.87E-08 |
|  | hsa-miR-99a | 10.49 | 3.22E-07 | 2.01E-06 |
|  | hsa-miR-221 | 6.55 | 2.11E-05 | 1.01E-04 |
|  | hsa-miR-671-3p | 6.06 | 1.92E-03 | 5.62E-03 |
|  | hsa-miR-222 | 4.97 | 6.24E-05 | 2.69E-04 |
|  | hsa-miR-193a-3p | 4.94 | 3.25E-03 | 8.72E-03 |
|  | hsa-miR-493 | 4.84 | 1.61E-04 | 6.49E-04 |
|  | hsa-miR-196b | 4.79 | 2.20E-03 | 6.20E-03 |
|  | hsa-miR-125b | 4.37 | 3.12E-03 | 8.46E-03 |
|  | hsa-miR-376a | 4.16 | 3.27E-03 | 8.72E-03 |
|  | hsa-miR-539 | 3.66 | 1.11E-03 | 3.34E-03 |
|  | hsa-miR-193b | 3.48 | 1.37E-03 | 4.04E-03 |
|  | hsa-miR-365 | 3.30 | 9.04E-04 | 2.79E-03 |
|  | hsa-miR-886-5p | 3.20 | 2.39E-02 | 4.95E-02 |
|  | hsa-miR-758 | 3.05 | 9.86E-03 | 2.34E-02 |
|  | hsa-miR-625 | 2.90 | 3.04E-04 | 1.14E-03 |
|  | hsa-miR-574-3p | 2.83 | 2.80E-04 | 1.08E-03 |
|  | hsa-miR-495 | 2.65 | 1.80E-02 | 3.90E-02 |
|  | hsa-miR-134 | 2.63 | 1.86E-02 | 4.01E-02 |
|  | hsa-miR-410 | 2.52 | 2.00E-02 | 4.25E-02 |
|  | hsa-miR-145 | 2.23 | 1.42E-02 | 3.17E-02 |
|  | hsa-miR-152 | 2.20 | 2.22E-02 | 4.64E-02 |
|  | hsa-miR-494 | 2.15 | 1.07E-02 | 2.48E-02 |
|  | hsa-miR-125a-5p | 1.58 | 1.96E-02 | 4.21E-02 |

In 40 ES biopsies 123 miRNAs with a FDR corrected q-value <0.05 were detected as differentially expressed compared to 6 MSC samples.
