# Supplementary material for: Differentially Expressed miRNAs in Ewing Sarcoma Compared to Mesenchymal Stem Cells: Low miR-31 Expression with Effects on Proliferation and Invasion
Source: PLoS One. 2014 Mar 25;9(3):e93067. doi: 10.1371/journal.pone.0093067 (PMC3965523; doi:10.1371/journal.pone.0093067)
Supplement: Table S3 — miRNAs differentially expressed comparing miRNA expression profiles generated with TLDAs of ES cell lines to MSCs. (DOCX) [file pone.0093067.s007.docx]

**Table S3.** miRNAs differentially expressed comparing miRNA expression profiles generated with TLDAs of ES cell lines to MSCs.

|  | miRNA | FC | p-value | q-value |
| --- | --- | --- | --- | --- |
|  |  |  |  |  |
| Higher expressed | hsa-miR-501-5p | 44.55 | 9.71E-04 | 3.21E-03 |
|  | hsa-miR-598 | 29.66 | 5.27E-04 | 1.93E-03 |
|  | hsa-miR-10b | 14.47 | 9.19E-06 | 1.94E-04 |
|  | hsa-miR-126 | 13.94 | 1.00E-06 | 6.34E-05 |
|  | hsa-miR-20b | 12.55 | 5.95E-04 | 2.13E-03 |
|  | hsa-miR-200c | 10.63 | 6.75E-03 | 1.76E-02 |
|  | hsa-miR-146b-5p | 9.30 | 5.49E-03 | 1.49E-02 |
|  | hsa-miR-17 | 7.53 | 6.10E-08 | 1.16E-05 |
|  | hsa-miR-106a | 7.15 | 4.40E-08 | 1.16E-05 |
|  | hsa-miR-301a | 6.93 | 1.55E-05 | 2.46E-04 |
|  | hsa-miR-340 | 6.62 | 1.03E-04 | 9.50E-04 |
|  | hsa-miR-652 | 5.48 | 1.07E-04 | 9.71E-04 |
|  | hsa-miR-301b | 5.46 | 1.93E-04 | 1.38E-03 |
|  | hsa-miR-18a | 5.41 | 1.34E-05 | 2.31E-04 |
|  | hsa-miR-20a | 5.00 | 7.93E-06 | 1.77E-04 |
|  | hsa-miR-18b | 4.90 | 9.99E-06 | 2.00E-04 |
|  | hsa-miR-331-5p | 4.82 | 1.54E-02 | 3.48E-02 |
|  | hsa-miR-92a | 4.67 | 3.35E-06 | 1.17E-04 |
|  | hsa-miR-19b | 4.52 | 6.02E-06 | 1.63E-04 |
|  | hsa-miR-19a | 4.28 | 2.38E-05 | 3.27E-04 |
|  | hsa-miR-128 | 3.94 | 7.79E-04 | 2.72E-03 |
|  | hsa-miR-422a | 3.87 | 4.01E-03 | 1.13E-02 |
|  | hsa-miR-93 | 3.67 | 6.79E-05 | 7.59E-04 |
|  | hsa-miR-345 | 3.65 | 9.49E-05 | 9.01E-04 |
|  | hsa-miR-342-3p | 3.51 | 5.59E-05 | 6.44E-04 |
|  | hsa-miR-192 | 3.18 | 8.49E-03 | 2.11E-02 |
|  | hsa-miR-296-5p | 3.18 | 7.46E-03 | 1.91E-02 |
|  | hsa-miR-186 | 3.02 | 1.42E-03 | 4.54E-03 |
|  | hsa-miR-362-5p | 2.91 | 2.81E-03 | 8.31E-03 |
|  | hsa-miR-454 | 2.89 | 2.43E-04 | 1.38E-03 |
|  | hsa-miR-374b | 2.63 | 3.37E-04 | 1.38E-03 |
|  | hsa-miR-106b | 2.62 | 2.68E-03 | 8.07E-03 |
|  | hsa-miR-130a | 2.50 | 4.58E-03 | 1.27E-02 |
|  | hsa-miR-324-5p | 2.48 | 5.53E-03 | 1.49E-02 |
|  | hsa-miR-491-5p | 2.32 | 1.90E-03 | 5.86E-03 |
|  | hsa-miR-532-5p | 2.19 | 2.01E-02 | 4.22E-02 |
|  | hsa-miR-331-3p | 2.12 | 6.47E-03 | 1.71E-02 |
|  | hsa-miR-590-5p | 1.93 | 2.39E-02 | 4.95E-02 |
|  | hsa-miR-324-3p | 1.88 | 9.87E-03 | 2.42E-02 |
|  | hsa-miR-484 | 1.60 | 1.86E-02 | 4.00E-02 |
|  |  |  |  |  |
| Lower expressed | hsa-miR-31 | 21106.12 | 1.66E-04 | 1.38E-03 |
|  | hsa-miR-143 | 2863.99 | 8.29E-05 | 8.51E-04 |
|  | hsa-miR-145 | 1387.69 | 2.05E-06 | 1.04E-04 |
|  | hsa-miR-886-5p | 1239.44 | 7.51E-05 | 8.16E-04 |
|  | hsa-miR-708 | 688.47 | 9.32E-05 | 9.01E-04 |
|  | hsa-miR-99a | 159.51 | 3.37E-05 | 4.41E-04 |
|  | hsa-miR-100 | 145.19 | 7.76E-06 | 1.77E-04 |
|  | hsa-miR-302b | 91.34 | 4.71E-04 | 1.79E-03 |
|  | hsa-miR-22 | 84.83 | 3.69E-06 | 1.17E-04 |
|  | hsa-miR-369-5p | 84.05 | 1.56E-03 | 4.91E-03 |
|  | hsa-miR-125b | 80.24 | 4.49E-05 | 5.50E-04 |
|  | hsa-miR-137 | 63.50 | 8.59E-03 | 2.12E-02 |
|  | hsa-miR-431 | 41.47 | 1.13E-02 | 2.74E-02 |
|  | hsa-miR-193a-5p | 36.44 | 2.47E-06 | 1.04E-04 |
|  | hsa-miR-138 | 29.81 | 8.14E-05 | 8.51E-04 |
|  | hsa-miR-654-3p | 29.41 | 1.82E-02 | 3.97E-02 |
|  | hsa-miR-34a | 29.14 | 6.75E-03 | 1.76E-02 |
|  | hsa-miR-152 | 27.94 | 1.54E-05 | 2.46E-04 |
|  | hsa-miR-654-5p | 26.60 | 1.71E-02 | 3.79E-02 |
|  | hsa-miR-574-3p | 23.89 | 8.56E-05 | 8.56E-04 |
|  | hsa-miR-376a | 21.65 | 5.08E-04 | 1.91E-03 |
|  | hsa-miR-193a-3p | 21.12 | 2.41E-05 | 3.27E-04 |
|  | hsa-miR-382 | 20.04 | 5.86E-03 | 1.57E-02 |
|  | hsa-miR-424 | 20.03 | 1.71E-02 | 3.79E-02 |
|  | hsa-miR-146a | 19.26 | 2.44E-02 | 4.98E-02 |
|  | hsa-miR-337-5p | 17.65 | 1.88E-02 | 4.00E-02 |
|  | hsa-miR-21 | 16.64 | 3.39E-06 | 1.17E-04 |
|  | hsa-miR-127-3p | 14.54 | 9.54E-04 | 3.18E-03 |
|  | hsa-miR-485-3p | 13.80 | 8.38E-04 | 2.87E-03 |
|  | hsa-miR-376c | 13.02 | 5.95E-04 | 2.13E-03 |
|  | hsa-miR-193b | 12.73 | 5.34E-05 | 6.34E-04 |
|  | hsa-miR-196b | 11.22 | 1.55E-02 | 3.48E-02 |
|  | hsa-miR-365 | 11.07 | 1.15E-05 | 2.09E-04 |
|  | hsa-miR-299-5p | 10.70 | 4.32E-04 | 1.67E-03 |
|  | hsa-miR-411 | 10.46 | 3.75E-04 | 1.52E-03 |
|  | hsa-miR-222 | 9.49 | 2.39E-06 | 1.04E-04 |
|  | hsa-let-7b | 9.45 | 3.95E-04 | 1.55E-03 |
|  | hsa-miR-379 | 9.30 | 8.49E-04 | 2.88E-03 |
|  | hsa-miR-655 | 8.93 | 3.10E-03 | 9.01E-03 |
|  | hsa-miR-671-3p | 8.84 | 6.82E-06 | 1.73E-04 |
|  | hsa-miR-493 | 8.77 | 2.64E-04 | 1.38E-03 |
|  | hsa-miR-134 | 8.59 | 3.29E-03 | 9.46E-03 |
|  | hsa-miR-221 | 8.57 | 5.10E-06 | 1.49E-04 |
|  | hsa-miR-27a | 8.49 | 3.85E-04 | 1.53E-03 |
|  | hsa-miR-370 | 6.60 | 5.03E-03 | 1.39E-02 |
|  | hsa-miR-29c | 6.19 | 6.45E-04 | 2.29E-03 |
|  | hsa-miR-494 | 6.08 | 5.28E-04 | 1.93E-03 |
|  | hsa-miR-495 | 5.56 | 7.41E-03 | 1.91E-02 |
|  | hsa-let-7d | 5.16 | 5.14E-04 | 1.91E-03 |
|  | hsa-miR-199a-3p | 5.09 | 2.65E-04 | 1.38E-03 |
|  | hsa-miR-758 | 4.96 | 1.89E-02 | 4.00E-02 |
|  | hsa-miR-29a | 4.67 | 1.26E-03 | 4.12E-03 |
|  | hsa-miR-29b | 4.67 | 7.96E-04 | 2.75E-03 |
|  | hsa-miR-625 | 4.62 | 8.59E-04 | 2.89E-03 |
|  | hsa-miR-27b | 4.41 | 2.73E-03 | 8.17E-03 |
|  | hsa-miR-24 | 4.17 | 2.22E-03 | 6.73E-03 |
|  | hsa-let-7a | 3.45 | 1.90E-03 | 5.86E-03 |
|  | hsa-let-7g | 3.43 | 1.21E-02 | 2.86E-02 |
|  | hsa-miR-140-5p | 3.30 | 3.58E-03 | 1.02E-02 |
|  | hsa-miR-140-3p | 3.01 | 1.39E-03 | 4.49E-03 |
|  | hsa-miR-28-5p | 2.65 | 1.94E-02 | 4.09E-02 |
|  | hsa-miR-214 | 2.49 | 3.00E-03 | 8.77E-03 |
|  | hsa-miR-28-3p | 2.15 | 1.87E-02 | 4.00E-02 |

In 6 ES cell lines 103 miRNAs with a FDR corrected q-value <0.05 are differentially expressed compared to 6 MSC samples.
