# Supplementary material for: Differentially Expressed miRNAs in Ewing Sarcoma Compared to Mesenchymal Stem Cells: Low miR-31 Expression with Effects on Proliferation and Invasion
Source: PLoS One. 2014 Mar 25;9(3):e93067. doi: 10.1371/journal.pone.0093067 (PMC3965523; doi:10.1371/journal.pone.0093067)
Supplement: Table S4 — Values from the apoptosis assays corresponding to Figure 2B. (DOCX) [file pone.0093067.s008.docx]

**Table S4.** Values from the apoptosis assays corresponding to Figure 2B.
