# Supplementary material for: Differentially Expressed miRNAs in Ewing Sarcoma Compared to Mesenchymal Stem Cells: Low miR-31 Expression with Effects on Proliferation and Invasion
Source: PLoS One. 2014 Mar 25;9(3):e93067. doi: 10.1371/journal.pone.0093067 (PMC3965523; doi:10.1371/journal.pone.0093067)
Supplement: Table S5 — Values from the cell cycle analysis corresponding to Figure 2C. (DOCX) [file pone.0093067.s009.docx]

**Table S5.** Values from the cell cycle analysis corresponding to Figure 2C.
