# Supplementary material for: Differentially Expressed miRNAs in Ewing Sarcoma Compared to Mesenchymal Stem Cells: Low miR-31 Expression with Effects on Proliferation and Invasion
Source: PLoS One. 2014 Mar 25;9(3):e93067. doi: 10.1371/journal.pone.0093067 (PMC3965523; doi:10.1371/journal.pone.0093067)
Supplement: Table S6 — Expression of miR-31 in MSCs and different cancer cell lines (ES, OS, breast cancer). (DOCX) [file pone.0093067.s010.docx]

**Table S6.** Expression of miR-31 in MSCs and different cancer cell lines (ES, OS, breast cancer).

| Sample | | Ct | dCt | FC |
| --- | --- | --- | --- | --- |
| MSC | 2 | 20.27 | 1.26 | 2.39 |
|  | 5 | 20.21 | 0.71 | 1.64 |
| ES | TC-71 | 35.00 | 17.69 | 211456.30 |
|  | RD-ES | 29.80 | 13.32 | 10190.94 |
|  | WE-68 | 26.72 | 9.48 | 711.64 |
|  | CADO-ES1 | 35.00 | 18.02 | 265803.39 |
|  | RM-82 | 26.51 | 9.33 | 641.36 |
|  | VH-64 | 35.00 | 17.18 | 148489.36 |
| OS | HOS | 22.44 | 5.19 | 36.38 |
|  | MG-63 | 19.53 | 0.98 | 1.98 |
| BC | MCF-7 | 32.72 | 14.83 | 29125.60 |
|  | MDA-MB-231 | 35.00 | 16.91 | 123145.13 |

For normalisation snRNA U6 was used.
