# Supplementary material for: Differentially Expressed miRNAs in Ewing Sarcoma Compared to Mesenchymal Stem Cells: Low miR-31 Expression with Effects on Proliferation and Invasion
Source: PLoS One. 2014 Mar 25;9(3):e93067. doi: 10.1371/journal.pone.0093067 (PMC3965523; doi:10.1371/journal.pone.0093067)
Supplement: Dataset S1 — Characterisation of mesenchymal stem cells. (DOC) [file pone.0093067.s011.doc]

**Dataset S1. Characterisation of mesenchymal stem cells.**

*Characteristics of the MSC preparations*

To validate the purity of the MSCs at the end of the in-vitro culture FACS-analyses were performed using the MSC-marker CD105 (PE-labelled) and the haematopoietic-cell specific marker CD45 (FITC-labelled). To verify the isolation and expansion of MSCs some cultured cells were tested for their potential to differentiate into adipocytes and osteoblasts using NH AdipoDiff or OsteoDiff Medium (Miltenyi Biotec Inc.). For detection of differentiation into adipocytes cells were stained with Oil Red O (Sigma-Aldrich, München, Germany). As osteoblasts express high levels of alkaline phosphatase, differentiation into osteoblasts was verified by stainings with SIGMA FAST BCIP/NBT substrate (Sigma-Aldrich).

*Unsupervised clustering of the six MSC preparations used for the TLDA analysis*


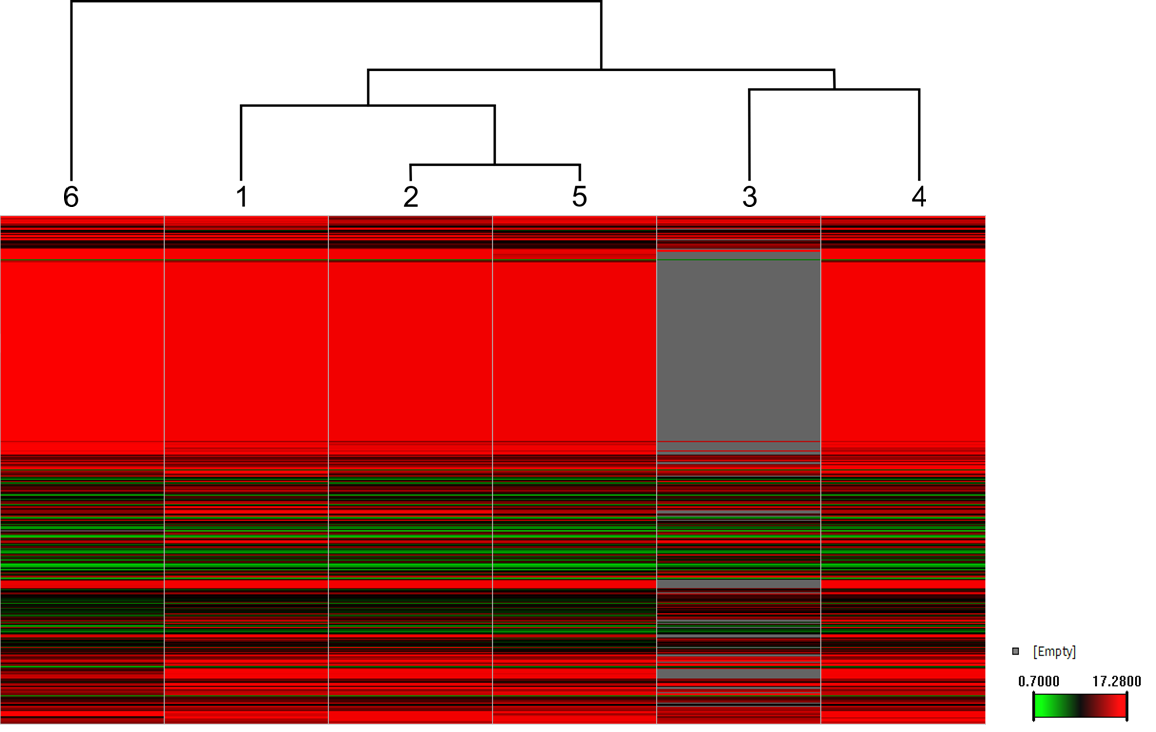


To assess the homogeneity of the MCS preparations regarding miRNA expression the six MSC-populations used for TLDAs were compared in unsupervised and supervised clustering analyses. For hierarchical clustering an unweighted average method and a Pearson correlation similarity measure were used. To identify significant differentially expressed miRNAs (q-value <0.05) within the MSC-group samples were divided into different subgroups. For all supervised comparisons (MSC 1-2-3 vs. MSC 4-5-6; MSC 1-4-5 vs. MSC 2-3-6; MSC 1-2-5 vs. MSC 3-4-6; MSC 1-3-4 vs. MSC 2-5-6; MSC 1-5-6 vs. MSC 2-3-4; MSC 1-2-5 vs. MSC 3-4-6) no significant differentially expressed miRNAs were detected.
