# Supplementary material for: Differentially Expressed miRNAs in Ewing Sarcoma Compared to Mesenchymal Stem Cells: Low miR-31 Expression with Effects on Proliferation and Invasion
Source: PLoS One. 2014 Mar 25;9(3):e93067. doi: 10.1371/journal.pone.0093067 (PMC3965523; doi:10.1371/journal.pone.0093067)
Supplement: Dataset S2 — Establishment of the transfection protocol. (DOC) [file pone.0093067.s012.doc]

**Dataset S2. Establishment of the transfection protocol.**

*Transfection efficiencies for ES cell lines using the HiPerFect transfection reagent*

Cells were transfected with 100 nM of a negative control siRNA labeled with Alexa Fluor 488. After 24 h fluorescent cells were counted and mean-values of three different fields of view were calculated.

*Dependence of siRNA mediated knock-down in ES cell lines on incubation time, transfection frequency and serum concentration*

To optimize the transfection conditions for miRNA mediated effects in ES cell lines, cells were transiently transfected with 100 nM of EZH2 siRNA or negative siRNA using HiPerFect reagent and immunoblots and the ImageJ-software were used to determine EZH2 expression relative to ß-actin. EZH2 was chosen because it is a direct EWSR1/FL1 target and strongly expressed in ES and as a siRNA with strong effects on EZH2 expression was available. EZH2 siRNA was only used to optimise the transfection conditions. (A) Time and transfection frequency dependence of EZH2 downregulation. Cells were cultured in medium containing 10% FCS and before each transfection cells were detached from the surface. Best efficiencies were obtained after three transfections with 72 h incubation. (B) FCS dependent effects on EZH2 downregulation. Cells were cultured for 72 h and transfected at time-points 0 h, 24 h and 48 h. Cells cultured in serum reduced medium showed a higher EZH2 reduction compared to cells cultured in medium containing 10% FCS. Additionally detaching cells prior to each transfection increased the efficiency. (C) Optimized transfection conditions shown as duplicates. For all examined cell lines the greatest EZH2 reduction was obtained after 72 h with transfections at time-points 0 h, 24 h and 48 h. TC-71 was cultured in 1% FCS; CADO-ES1, WE-68 and RD-ES required medium with 3% FCS.
